# Supplementary material for: A structured medication review tool to promote psychotropic medication optimisation for adults with intellectual disability: feasibility study
Source: BMJ Open. 2019 Dec 15;9(12):e033827. doi: 10.1136/bmjopen-2019-033827 (PMC6924735; doi:10.1136/bmjopen-2019-033827)
Supplement: Author's manuscript [file bmjopen-2019-033827.draft_revisions.pdf]

BMJ Open is committed to open peer review. As part of this commitment we make the peer review history of every article we publish publicly available.

When an article is published we post the peer reviewers' comments and the authors' responses online. We also post the versions of the paper that were used during peer review. These are the versions that the peer review comments apply to.

The versions of the paper that follow are the versions that were submitted during the peer review process. They are not the versions of record or the final published versions. They should not be cited or distributed as the published version of this manuscript.

BMJ Open is an open access journal and the full, final, typeset and author-corrected version of record of the manuscript is available on our site with no access controls, subscription charges or pay-per-view fees (http://bmjopen.bmj.com).

If you have any questions on BMJ Open's open peer review process please email info.bmjopen@bmj.com

BMJ Open

A structured medication review tool to promote psychotropic medication optimisation for adults with intellectual disability: feasibility study

Journal:	*BMJ Open*
Manuscript ID	bmjopen-2019-033827
Article Type:	Original research
Date Submitted by the Author:	23-Aug-2019
Complete List of Authors:	Sheehan, Rory; University College London, Division of Psychiatry<br>Strydom, André; Institute of Psychiatry Psychology and Neuroscience<br>Marston, Louise; University College London, Research Department of Primary Care and Population Health<br>Morant, Nicola; University College London, Division of Psychiatry<br>Fiori, Federico; Institute of Psychiatry, Psychology & Neuroscience, Department of Child and Adolescent Psychiatry<br>Santosh, Paramala; King's College London , Department of Child and Adolescent Psychiatry<br>HASSIOTIS, ANGELA; University College London, Division of Psychiatry
Keywords:	intellectual disability, psychotropic medication, medication optimisation, medication review, feasibility study

SCHOLARONE™
Manuscripts

TITLE PAGE

A structured medication review tool to promote psychotropic medication optimisation for adults with intellectual disability: feasibility study

Dr Rory Sheehan, MSc, Division of Psychiatry, University College London, London, UK

Professor André Strydom, PhD, Institute of Psychiatry, Psychology and Neuroscience, King's College London, London, UK

Dr Louise Marston, PhD, Research Department of Primary Care and Population Health, Division of Psychiatry, University College London, London, UK

Dr Nicola Morant, PhD, Division of Psychiatry, University College London, London, UK

Dr Federico Fiori, Institute of Psychiatry, Psychology and Neuroscience, King's College London, London, UK

Professor Paramala Santosh, Institute of Psychiatry, Psychology and Neuroscience, King's College London, London, UK

Professor Angela Hassiotis, PhD, Division of Psychiatry, University College London, London, UK

Corresponding author details

Dr Rory Sheehan

Division of Psychiatry, University College London, 6th Floor Maple House, 149 Tottenham Court Road, London W1T 7NF

Telephone: 0207-679-9201

E-mail: r.sheehan@ucl.ac.uk

For peer review only

For peer review only - http://bmjopen.bmj.com/site/about/guidelines.xhtml

Word count

3,851

Number of figures and tables

2 tables

3 figures

Keywords

Intellectual disability; psychotropic medication; medication optimisation; medication review; feasibility study

For peer review only

A structured medication review tool to promote psychotropic medication optimisation for adults with intellectual disability: feasibility study

ABSTRACT

Objectives To investigate the feasibility of delivering structured psychotropic medication review in community services for adults with intellectual disability.

Design Single-arm feasibility study conducted over a six-month period.

Setting Specialist community intellectual disability teams in England.

Participants Psychiatrists working with adults with intellectual disability and adults with intellectual disability who had been prescribed psychotropic medication.

Intervention A structured web-based psychotropic medication review tool (the HealthTracker™-based structured medication review) comprising measures of therapeutic benefit and adverse side-effects was made available for use by psychiatrists in routine clinic appointments. A summary measure of medication effectiveness was graphically presented to aid decision-making.

Main outcome measures Feasibility metrics including number of people referred, eligible, and recruited, and uptake of the medication review tool in naturalistic clinical settings.

For peer review only - http://bmjopen.bmj.com/site/about/guidelines.xhtml

Participant feedback was collected to assess acceptability of the intervention and suggestions for development.

Results Fifteen psychiatrists from five clinical teams took part. In total 94 potentially-eligible participants were referred, of whom 79 (84%) were recruited and together underwent 97 medication reviews over the six month study period. Feedback from participants with intellectual disability was favourable. Psychiatrists indicated the HealthTracker™-based medication review was broadly acceptable and indicated adaptations to improve integration with existing information technology systems and to enhance patient involvement in the review.

Conclusions Structured psychotropic medication review can be used in community services for adults with intellectual disability as part of a programme of medication optimisation. It would be feasible to test clinical and patient outcomes of the HealthTracker™-based medication review in a randomised clinical trial.

For peer review only - http://bmjopen.bmj.com/site/about/guidelines.xhtml

STRENGTHS AND LIMITATIONS OF THIS STUDY

- This feasibility study is one of the first to suggest a pragmatic and scalable means of achieving psychotropic medication optimisation in people with intellectual disability using structured medication review.
- The work provides estimates of recruitment rate and uptake of the intervention, as well as suggestions for its development, that can inform the planning and delivery of a future clinical trial.
- The study was conducted in a single region of the UK which may not be representative of other locations or healthcare settings.
- Details of those who were eligible but did not participate in the study were not collected and the overall rate of uptake of the intervention cannot be determined.

For peer review only - http://bmjopen.bmj.com/site/about/guidelines.xhtml

INTRODUCTION

Intellectual disability (ID), present in approximately 2% of the population, is a lifelong disorder defined by significant cognitive deficit and impaired functional and adaptive skills.[1] Between a third and one half of adults with ID are prescribed psychotropic medication.[2, 3] Renewed focus on the quality of prescribing has been prompted by epidemiological evidence which shows that the extent of psychotropic use is disproportionate to prevalence of mental illness in this group, and medication is often used 'off-label' in the management of behaviour that challenges.[4] People with ID are at greater risk of idiosyncratic reactions and adverse medication side-effects than their non-intellectually disabled counterparts and are more likely to receive high psychotropic doses, polypharmacy, and to remain on psychotropic medication for extended periods.[5, 6]

The UK Government has committed to improving the use of psychotropic medication in people with ID[7] and a national programme, Stopping the Over-Medication of People with Learning Disabilities (STOMP), was established in 2016 to raise awareness of the issue and stimulate activity amongst patients, advocates, and professionals.[8] Medication optimisation is a multi-faceted concept that aims to promote the best use of prescribed medication by prioritising safety, evidence-based choice of medication, and centring patient experience and involvement.[9] Medication review, a structured and critical evaluation of a prescribed medication, is a key element of medication optimisation that is recommended by the National Institute for Health and Care Excellence (NICE) for groups at high risk of suboptimal medication use.[10] Structured medication review offers a number of potential benefits including: promoting systematic evaluation of desired and undesired medication effects;

For peer review only - http://bmjopen.bmj.com/site/about/guidelines.xhtml

standardisation of assessment across time and between clinicians; an efficient method of recording information; and making explicit the basis on which decisions are made. A recent systematic review found that psychotropic medication review is associated with change or reduction in number of drugs prescribed but consistent improvement in clinical and patient-reported outcomes has not been shown, and there is considerable variation and little formal guidance on how medication reviews are operationalised.[11] We undertook a study to investigate the feasibility of a structured psychotropic medication review (the HealthTracker™-based structured medication review, HT-SMR) in community psychiatry of ID teams. Specific objectives were to determine how many clinicians and patients could be recruited, to assess the uptake of this novel intervention in real-world clinical settings, and to gather feedback that could inform future development and refinement of the intervention.

METHOD

Study procedures

This was a single-arm feasibility study conducted over a six-month period in five community psychiatry of ID services in London, UK. All services were part of the National Health Service. Adults (>18 years) with ID were eligible to participate if they were prescribed psychotropic medication of any type and for any indication. Psychiatrists were asked to briefly introduce the research to potential participants and/or their carers, either at routine appointments or by sending an information leaflet through the post. The contact details of those who

For peer review only - http://bmjopen.bmj.com/site/about/guidelines.xhtml

expressed interest were passed to the research team who then met with the potential participant to explain the research in more detail and confirm eligibility. Written informed consent was obtained from all participants. Ability to consent to take part was assessed according to the principles of the Mental Capacity Act.[12] If a person lacked capacity to consent, a family member or nominated consultee was sought to give advice to the research team on the person's inclusion. All study materials were available in accessible (easy-read) format. When a participant was recruited to the study, their psychiatrist was informed and was then able to use the HT-SMR in appointments during the study period.

Intervention

The intervention consisted of the HT-SMR (figure 1) designed to be used in routine clinical appointments by a participant's psychiatrist. The HealthTracker™ is a password-protected web-based health monitoring platform that originated in the NHS, with the NHS receiving royalties from its use. For the purposes of this study, medication review included a record of basic demographic, clinical and treatment information along with responses to the Profile of Treatment Response (POTR). The POTR comprises two generic scales; one measuring therapeutic response to a medication over several symptom domains, the other measuring potential adverse side-effects. Each item is rated by the psychiatrist on a Likert scale using information gathered from observation and the clinical interview. Items that are not applicable can be marked as such but incomplete reviews cannot be submitted. Based on responses to the two scales above, the HealthTracker™ imputes the Modified Efficacy Index (MEI) as the ratio between the therapeutic benefit of a medication and the presence of adverse side-effects. The MEI is then displayed in a simple colour-coded matrix that allows

For peer review only - http://bmjopen.bmj.com/site/about/guidelines.xhtml

the clinician to see how the patient has responded to treatment and may act as a stimulus for discussion with the patient and/or carer. The Clinical Global Impression-Improvement (CGI-I), a well-established rating tool that can be completed quickly and easily in clinical settings,[13] is completed by the psychiatrist for each medication as a further measure of medication effect. We asked psychiatrist to record if they had advised a change to medication following the review.

FIGURE 1 NEAR HERE - HealthTracker™-based structured medication review (HT-SMR)

Each participant was assigned a unique identification number and pseudonymised data collected in the medication review were stored on a secure electronic cloud. A single medication or multiple medications could be reviewed at one time, with a separate POTR for each drug. If the HT-SMR is used across different time periods, a longitudinal record of treatment response is generated. The researcher trained the participating psychiatrists on the system in a face-to-face session and was available throughout the study for support as needed.

Data from medication reviews were downloaded from the HealthTracker™ as a CSV file into an SPSS database at the end of the study period. The POTR, MEI and CGI-I results were summarised with descriptive statistics. Spearman's correlation between the MEI and CGI-I and the psychiatrist's decision to change or not change medication were calculated. Owing to the skewness of the data, non-parametric tests were used to test the significance of associations.

For peer review only - http://bmjopen.bmj.com/site/about/guidelines.xhtml

For peer review only

Feasibility measures

We gauged interest from clinical teams and individual psychiatrists to take part in the study and recorded the rates of referral and recruitment of participants, and of uptake of the medication review tool in routine clinic appointments. Reasons for not recruiting people who were referred to the research team were noted. As this was a feasibility study, a formal sample size calculation was not performed but our *a priori* estimate was that 100 people would be recruited, based on previous feasibility studies that have trialled similar interventions in community settings.[14]

Participant characteristics

Characteristics of participants and descriptive data are reported. Medication doses were converted to defined daily dose (DDD).[15]

Acceptability and implementation

At the end of each medication review, psychiatrists asked participants with ID, “How able were you to say everything you wanted to say about medication today?” Answers were scored on a five-point Likert scale with pictorial cues alongside the response set to improve understanding. At the end of the study period, psychiatrists were invited to complete an anonymous web-based survey designed for this study with a mix of closed and open-ended questions. The survey concerned the research process, experience and views on use of the

For peer review only - http://bmjopen.bmj.com/site/about/guidelines.xhtml

online review system, and suggested adaptations to maximise usability and utility of the medication review in its future development. Responses to the psychiatrist feedback questionnaire were summarised in a structured analysis within pre-determined categories. All data were managed in SPSS v.24 and Microsoft Excel.

Patient and public involvement

A service user consultation group was formed as part of the broader work within which this study was conducted. The consultation group consisted of people with ID and experience of medication use. We held regular meetings with the consultation group, who advised on various aspects of this work including, the recruitment strategy, participant materials and easy-read information, devising the outcome measure for participants with ID, and general advice to aid successful conduct of the research. The group will be involved in dissemination to a broad range of relevant stakeholders.

Ethical approval

The authors assert that all procedures contributing to this work comply with the ethical standards of the relevant national and institutional committees on human experimentation and with the Helsinki Declaration of 1975, as revised in 2008. All procedures involving patients were approved by the London Bridge Research Ethics Committee (ref: 18/LO/1112).

RESULTS

For peer review only - http://bmjopen.bmj.com/site/about/guidelines.xhtml

Recruitment and uptake

Five community ID teams comprising fifteen psychiatrists were invited and agreed to take part in the feasibility study. Together, 94 people with ID were referred as potential participants over the six-month study period and 79 (84%) were recruited. Psychiatrists used the online system for medication review in 68 people (86% of those recruited). A number of people (*n*=21) had more than one medication review giving a total of 97 HT-SMRs (figure 2).

FIGURE 2 NEAR HERE - Participant flow

There was a steady state of referral, recruitment and review tool use (figure 3a). Recruitment and uptake of the HT-SMR was unequal between participating community ID teams and not related to the number of psychiatrists in each of these teams (figure 3b). No harms or unintended consequences were reported during the study and no participants withdrew their consent.

FIGURE 3 NEAR HERE – a) Rate of referral, recruitment, and use of the HT-SMR over the study period, and b) by participating clinical team

Participant information and data from medication reviews

Demographic data of participants who had medication review are summarised in table 1. The group was relatively young and most had mild ID. A primary diagnosis was not recorded

For peer review only

in just over half of the participants; in these cases it is possible that psychotropic medication was prescribed for behaviour that challenges.

Table 1 Demographic characteristics of study participants

**Characteristic**	***n* (%)**
**Sex**	
Male	41 (60%)
Female	27 (40%)
**Age at first HT-SMR (years)**	
18-25	23 (34%)
26-35	16 (24%)
36-45	8 (12%)
46-55	17 (25%)
55-65	1 (1%)
>65	3 (4%)
**Degree of ID**	
Mild	42 (62%)
Moderate	18 (26%)
Severe-profound	8 (12%)
**Ethnicity**	
White	35 (51%)
Black	14 (21%)
Asian	10 (15%)
Mixed / other	7 (10%)
Not known / not given	2 (3%)
**Primary diagnosis**	
Schizophrenia spectrum disorder	12 (18%)
Mood disorder	5 (7%)
Anxiety disorder	3 (4%)

For peer review only - http://bmjopen.bmj.com/site/about/guidelines.xhtml

Personality disorder	1 (1%)
Pervasive developmental disorder	7 (10%)
Attention deficit hyperactivity disorder	3 (4%)
Missing	37 (54%)

Of the 97 HT-SMRs conducted using the system, the most commonly reviewed drug class was antipsychotics (49 reviews), followed by anti-depressants (28 reviews) (table 2). The median prescribed dose of medication reviewed was 100% DDD (inter-quartile range, IQR, 50-133%) and median duration of use was 18 months (IQR, 5-56 months). Following the HT-SMR, psychiatrists advised a change to medication in just over one-third (*n*=27, 36%) cases.

Table 2 Summary results from the HT-SMRs (*n*=97 reviews)

Drug class reviewed	Number of reviews (% of all reviews)	Median DDD of medication reviewed (IQR)	Median duration of use (months) (IQR)	Median CGI-Improvement (IQR)*	Median Modified Efficacy Index (IQR)†
**Antipsychotic**	49 (51%)	67 (45-100)	24 (4-60)	1.5 (1.0-2.0)	2.0 (1.0-3.0)
**Anti-depressant**	28 (29%)	150 (87-200)	12 (4-24)	2.0 (1.0-3.0)	2.0 (1.4-3.0)
**Anxiolytic / sedative**	9 (9%)	24 (2-54)	100 (39-100)	3.0 (2.0-3.0)	3.0 (2.3-4.0)
**Medication for ADHD**	9 (9%)	120 (78-138)	18 (12-78)	2.0 (1.0-2.5)	2.0 (1.0-4.0)
**Mood stabiliser**	2 (2%)	33	96	2.5	1.2

For peer review only - http://bmjopen.bmj.com/site/about/guidelines.xhtml

**All**	97 (100%)	100 (50-133)	18 (5-56)	2.0 (1.0-3.0)	1.5 (1.0-3.0)

IQR, inter-quartile range

*CGI is scored between 1 (very much improved) and 7 (very much worse)

†Modified Efficacy Index is the ratio between the score in the domain with the greatest therapeutic benefit to the score in the domain with the worst rated adverse side-effect. Higher scores indicate more favourable medication response.

The HealthTracker™ imputed MEI can take a value of 0.33 to 4.0, where higher values equate to a more favourable therapeutic effect:adverse side-effect ratio. The median HealthTracker™ imputed MEI for medications reviewed was 1.5 (IQR 1.0-3.0). There was a statistically-significant negative correlation between the MEI and the CGI-I (where a lower score indicates greater perceived benefit of medication) (ρ -0.296, p=0.024; indicating 'fair' correlation between the two measures).[16] The MEI was significantly lower in those who had a medication change made following the review (median MEI 1.0, IQR 0.67-2.0) compared with those in whom no medication change was made following the review (median MEI 1.5, IQR 1.3-3.0) (p=0.011).

Acceptability and implementation

When asked "How able were you to say everything you wanted to say about medication today?", participants with ID responded "very easy" or "easy" in 54 (70%) cases, "not easy or difficult" in 14 (18%) cases, "difficult" or "very difficult" in 1 (1%) case. The question was not answered by 9 (12%) participants.

For peer review only - http://bmjopen.bmj.com/site/about/guidelines.xhtml

Fourteen psychiatrists out of fifteen completed the online feedback questionnaire. Results are presented as major themes with anonymised quotations to illustrate points of interest.

Feedback about the recruitment process

Although the majority (13/14) of psychiatrists reported that it had been ‘easy’ to introduce the study to potential participants, most (11/14) had encountered barriers. The main barriers to recruitment were *“time constraints”* within appointments and difficulties explaining the research to potential participants, especially those with more severe ID.

Psychiatrists were asked if the people who did not wish to hear more about the research had given reasons for their decision. The most commonly reported reason was worry about the commitment or inconvenience the research would entail. Others declined to hear more as they were already taking part in research or were content with their current medication regimen and did not want to discuss this further. Several psychiatrists reported that the person’s carer had not wished to pursue the research opportunity, either because they felt it was not appropriate or because they were not willing to act as a consultee in cases where the person with ID was likely to lack capacity to provide informed consent.

Ease of using the HealthTracker™ online system

For peer review only - http://bmjopen.bmj.com/site/about/guidelines.xhtml

Twelve psychiatrists reported having used the online system for medication review. In response to the question, “How easy was it to use the HealthTracker?” only one person reported it was “difficult”; the majority said it was “easy” or “very easy”.

Benefits of HT-SMR

Eight out of 12 psychiatrists were of the opinion that using the online medication review had helped people with ID or their carer to be more involved in the discussion about medication and promoted *“collaborative decision-making”*. Psychiatrists commented that it had been *“helpful as a template”* in *“framing the discussion around medication”* and that its use facilitated *“more in-depth”* and *“comprehensive”* medication review. The transparency of medication review using the system was described as an advantage: *“[using the tool] was an eye-opener for the patient and carer. They could hear the specific questions being asked systematically and seeing the matrix [the graphical representation of the EI] was really useful, particularly for a few participants who were not clear about medication”*.

Disadvantages of the HT-SMR

Several psychiatrists described logistical problems in using a system which required internet connectivity e.g. computers not working or running fast enough, lack of internet access in clinic settings, not having portable devices for domiciliary visits. Using the system took additional time which was sometimes difficult to find in the regular appointment.

For peer review only - http://bmjopen.bmj.com/site/about/guidelines.xhtml

Just over half of psychiatrists expressed the view that using the online medication review had interfered with their interaction with the patient or carer with one remarking that they had spent *"more time focussed on the computer rather than face-to-face personal interaction"*. A small number considered the system too rigid and resisted the *"imposed structure"* of the medication review which they believed was not always aligned with the patient's most pressing concerns.

Effect on decision-making

Eight out of 12 psychiatrists thought that undertaking the HT-SMR had helped them to make a decision about medication and 5/12 considered the tool made it more likely they would change medication compared with their usual practice. However in the survey free-text responses most commented that the medication review did not cause them to change decisions they would ordinarily have made, rather, the HT-SMR was viewed as *"an additional tool"* which could *"confirm a clinical impression," "justify decisions"* and give clinicians *"more confidence"*.

Adaptations and views about future use

Eight out of 12 psychiatrists thought that SMR should be used more widely. Suggestions to improve the system centred on making the system more *"user friendly"* and *"intuitive"* for psychiatrists, and integrated with existing computerised systems. Some also mentioned improving the accessibility to people with ID incorporating their views more formally in the

For peer review only

medication review e.g. *"adding a weight to the [decision-support] algorithm based on patient preference"*.

DISCUSSION

Main findings

There is a need to improve the quality of psychotropic medication use in people with ID yet despite consensus guidelines of good practice,[17, 18] there has been relatively little work to investigate practical methods to achieve medication optimisation in this group. The current study introduced a structured medication review tool in community psychiatric services for adults with intellectual disability and demonstrates that it would be feasible to test outcomes in a definitive trial.

Perhaps owing to the scrutiny currently applied to psychotropic prescribing, clinical teams and psychiatrists that we approached were keen to take part. Recruitment of people with ID to research can be challenging[19] but the number of participants we recruited was satisfactory, close to our original broad expectation, and the referral:recruitment ratio was high, indicating the processes of participant identification, recruitment and consent were appropriate.

A key question was whether psychiatrists were able and willing to integrate use of the HT-SMR into their standard practice, given the demands on their time and numerous mandated clinical and administrative tasks. Uptake of the HT-SMR was good, though not universal;

For peer review only - http://bmjopen.bmj.com/site/about/guidelines.xhtml

three psychiatrists did not use the tool and eleven people with ID who were recruited did not have a recorded medication review. The rate of missed appointments is higher in psychiatric clinics than in other medical specialties[20] and may be higher still in ID services and missed appointments are one likely cause that limited the HT-SMR during the study period.

The HealthTracker™ imputed MEI was tested as a potential future outcome measure. The MEI was correlated with the overall CGI-I and was lower (indicating a less favourable risk:benefit ratio) in those in whom medication changes were made compared with those in whom medication remained unchanged. The HealthTracker™ imputed MEI showed sufficient variation between participants and had value as a practical support to psychiatrists in considering medication changes, though the survey data showed that this does not replace psychiatrists' clinical judgement.

This research involved relatively little commitment from participants with ID and the intervention appeared acceptable in view of the recruitment metrics and response to the evaluation questionnaire. Two-thirds of psychiatrists thought the system should be used more extensively, indicating an overall favourable attitude. In order to maintain proximity to usual practice, we gave psychiatrists flexibility and few instructions of how to use the online system, other than how to enter data. There was clearly variation in how different psychiatrists approached the HT-SMR; positive feedback showed that some appreciated the systematic and comprehensive nature of the medication review and believed that it could facilitate a discussion with the person with ID. Negative comments referred to the perception that the structured review was inflexible and rigid. This may be related to natural

For peer review only - http://bmjopen.bmj.com/site/about/guidelines.xhtml

variation in clinicians' consultation style and familiarity with incorporating standardised or structured elements to the consultation, although these are recommended in monitoring medication effects.[18, 21]

Some psychiatrists reported disruption to the relational aspects of the consultation arising from the need to interact simultaneously with the computer screen and the person with ID and others who may attend the appointment. Electronic records are already used extensively in healthcare settings but use of technology as a more dynamic application may represent a more profound culture change and requires the development of new skills and ways of working. It is possible that digital interventions, if properly designed, can enhance communication between doctor and patient, for example, by incorporating augmentative and alternative communication methods.[22, 23] Given that patient involvement and the opportunity for shared decision-making is fundamental to medication optimisation, the HT-SMR would benefit from incorporating a greater role for people with ID and their carers to amplify the patient voice. This could go some way to countering the lack of involvement that patients and their carers often describe when medication decisions are made.[24] Other opportunities to extend the remit of this system include patients or carers completing measures in advance of appointments in order to release consultation time for discussion and collaboration.

Future work

A future clinical trial is needed to test if use of the HT-SMR contributes to medication optimisation. The HealthTracker™ imputed MEI could be used as a primary outcome

For peer review only - http://bmjopen.bmj.com/site/about/guidelines.xhtml

measure and should be supplemented by other measures of medication optimisation, including service utilisation, medication safety incidents, and patient-reported outcomes, including decision self-efficacy and satisfaction. An economic evaluation is also necessary to determine the cost implications of the intervention; balanced against the additional resource and infrastructure necessary to deliver the HT-SMR are potential cost savings achieved through reductions in medication waste and in indirect costs related to adverse side-effects.

Widescale implementation of a system of structured medication review would create a powerful naturalistic dataset of medication use, therapeutic impact, and adverse side-effects that could be used both as a dashboard to monitor and benchmark prescribing practice, and for observational research in this group where there is a paucity of empirical data and little prospect of significant future controlled trials.

The STOMP campaign in England has so far not achieved discernible reductions in psychotropic prescribing to adults with ID.[25] Medication review, as an opportunity for critical reflection and discussion about medication, may act as a stimulus for change in prescribing that will ultimately improve medication outcomes. However, there are many influences on prescribing behaviour, including those acting on an individual level amongst patients, carers and clinicians,[26, 27] as well as systemic factors which are likely to extend beyond the control of the prescriber, such as appropriately-supported accommodation and social care provision.[28] Thus, a medication review intervention can only be one element of a programme of medication optimisation and changing behaviour on a wider scale will require concerted action across health and social care sectors. One published report of a multi-

For peer review only - http://bmjopen.bmj.com/site/about/guidelines.xhtml

component intervention to reduce antipsychotic use has shown some success but was time-consuming, has not been replicated, and lacks longer-term outcomes.[29] Future evidence-based complex interventions (of which structured medication review can be a part) that can work at scale should be underpinned by a theoretical framework that can identify the levers and barriers that are most likely to affect implementation.[30]

Strengths and limitations of this study

This study was completed in real-world settings, included psychiatrists of different grades from several different services, and a diverse group of participants, thereby increasing generalisability of the findings. We obtained estimates of important recruitment parameters and confirmed a successful recruitment strategy. Feedback has enabled us to identify aspects of the HT-SMR which require development to improve utility and enhance the potential for benefits of the intervention. The advantages of this mediation review were that it is relatively quick, self-explanatory, and can be completed in a single patient contact, making it easier to integrate into the current models of care than other published medication review methods that are multi-stage and multi-professional and more likely to encounter implementation barriers.[31, 32] Being conducted by the psychiatrist, who is also the prescriber, avoids the pitfalls of non-prescriber directed medication reviews in which as few as one-third of recommendations are actioned.[33]

This relatively small-scale study also had limitations. We could not collect the characteristics of those who declined to participate in the research, and therefore do not know the total eligible population or whether certain groups were under-represented in our sample.

For peer review only - http://bmjopen.bmj.com/site/about/guidelines.xhtml

Similarly, we do not know the number of appointments in which the system could have been used in but was not, and without this denominator cannot report the rate of uptake. Attrition and clinician fatigue in using the online medication review may be an issue in a longitudinal study that was not addressed in this feasibility study, given the relatively short time-period of the research. A single participant feedback question was chosen to minimise demands placed on participants but was inevitably limited in scope and responses may have been subject to social desirability bias. Although logic suggests that the medication review would give patients and carers a greater opportunity for input in the process of medication decision-making, this was not formally tested and there was no method for gathering feedback from carers who may have been involved in the appointment and who play an important role in the medication process.

Conclusion

Medication review has potential to improve individual medication outcomes as part of a wider programme of medication optimisation. The HT-SMR could be tested in a definitive trial after some refinement to improve integration with existing software and to fully embed patient and carer voice in the review process.

For peer review only - http://bmjopen.bmj.com/site/about/guidelines.xhtml

Acknowledgements

The authors would like to thank staff in the clinical services who recruited participants and completed the HT-SMR, members of the Camden Synergy service user consultation group (Jill Huntesmith and Jackie McMorrow), and the research participants and their carers.

Author contribution statement

All authors conceived and designed the study and contributed towards the conduct and management of the work. RS recruited participants. RS, FF, and LM managed and analysed the data and all authors supervised this process. All authors interpreted the results. RS wrote the first draft of the paper and all authors contributed to further drafts and read and approved the final manuscript.

Funding

RS is supported by a Doctoral Research Fellowship from the National Institute for Health Research (NIHR) (Ref: DRF-2016-09-140).

Disclaimer

The views expressed in this article are those of the authors and not necessarily those of the NIHR or the Department of Health and Social Care. The funder had no role in study design, analysis, decision to publish, or preparation of the manuscript.

For peer review only - http://bmjopen.bmj.com/site/about/guidelines.xhtml

Data sharing

No additional data are available.

Competing interests

All authors have read and completed the ICMJE form for competing interests.

Dr Sheehan reports no conflict of interest.

Professor Strydom reports no conflict of interest.

Dr Marston reports no conflict of interest.

Dr Morant reports no conflict of interest.

Dr Fiori reports that he is employed as Chief Technology Officer by HealthTracker Ltd - the company that has the copyright for the HealthTracker^{TM}-based Structured Medication Review (HT-SMR). The HT-SMR can be licensed to hospitals and healthcare settings.

Professor Santosh reports that he is a Director and shareholder of HealthTracker Ltd - the company that has the copyright for the HealthTracker^{TM}-based Structured Medication Review (HT-SMR). The HT-SMR can be licensed to hospitals and healthcare settings.

Professor Hassiotis reports no conflict of interest.

For peer review only - http://bmjopen.bmj.com/site/about/guidelines.xhtml

REFERENCES

1. American Psychiatric Association, editors. *Diagnostic and statistical manual of mental disorders: DSM-5)*: American Psychiatric Association, 2013.
2. Tsiouris JA, Kim S-Y, Brown WT, Pettinger J, Cohen IL. Prevalence of psychotropic drug use in adults with intellectual disability: positive and negative findings from a large scale study. *J Autism Dev Disord* 2013;**43**(3):719-31.
3. Bowring D, Totsika V, Hastings R, Toogood S, McMahon M. Prevalence of psychotropic medication use and association with challenging behaviour in adults with an intellectual disability. A total population study. *J Intellect Disabil Res* 2017;**61**(6):604-17.
4. Sheehan R, Hassiotis A, Walters K, Osborn D, Strydom A, Horsfall L. Mental illness, challenging behaviour, and psychotropic drug prescribing in people with intellectual disability: UK population based cohort study. *BMJ* 2015;**351**:h4326.
5. Straetmans JM, van Schrojenstein Lantman-de HM, Schellevis FG, Dinant G-J. Health problems of people with intellectual disabilities: the impact for general practice. *Br J Gen Pract* 2007;**57**(534):64-6.
6. De Kuijper G, Hoekstra P, Visser F, Scholte F, Penning C, Evenhuis H. Use of antipsychotic drugs in individuals with intellectual disability (ID) in the Netherlands: prevalence and reasons for prescription. *J Intellect Disabil Res* 2010;**54**(7):659-67.
7. NHS England. *NHS Long Term Plan.* NHS England, 2019 (https://www.longtermplan.nhs.uk)
8. Branford D, Gerrard D, Saleem N, Shaw C, Webster A. Stopping over-medication of people with an intellectual disability, autism or both (STOMP) in England part 2–the story so far. *Advances in Mental Health Intellectual Disabilities* 2019;**13**(1):41-51.
9. Royal Pharmaceutical Society. *Medicines Optimisation: helping patients to make the most of medicines.* Royal Pharmaceutical Society, 2013.
10. National Institute for Health and Care Excellence. *Medicines optimisation: the safe and effective use of medicines to enable the best possible outcomes. NICE Guideline NG5.* National Institute for Health and Care Excellence, 2015.

For peer review only

For peer review only - http://bmjopen.bmj.com/site/about/guidelines.xhtml

11. Sheehan R, Strydom A, Brown E, Marston L, Hassiotis A. Association of focused medication review with optimization of psychotropic drug prescribing: a systematic review and meta-analysis. *JAMA Network Open* 2018;**1**(6):e183750.

12. Mental Capacity Act 2005. The Stationery Office, 2005.

13. Busner J, Targum SD. The clinical global impressions scale: applying a research tool in clinical practice. *Psychiatry (Edgmont)* 2007;**4**(7):28.

14. Moncrieff J, Azam K, Johnson S, Marston L, Morant N, Darton K, et al. Results of a pilot cluster randomised trial of the use of a Medication Review Tool for people taking antipsychotic medication. *BMC Psychiatry* 2016;**16**(1):205.

15. World Health Organization. ATC/DDD Toolkit. World Health Organisation (https://www.who.int/medicines/regulation/medicines-safety/toolkit_ddd/en/)

16. Chan Y. Biostatistics 104: correlational analysis. *Singapore Med J* 2003;**44**(12):614-9.

17. Shankar R, Wilcock M, Oak K, McGowan P, Sheehan R. Stopping, rationalising or optimising antipsychotic drug treatment in people with intellectual disability and/or autism. *Drug Ther Bull* 2019;**57**(1):10-3.

18. Royal College of Psychiatrists Faculty of Psychiatry of Intellectual Disability. *Psychotropic drug prescribing for people with intellectual disability, mental health problems and/or behaviours that challenge: practice guidelines.* Royal College of Psychiatrists, 2016.

19. Mulhall P, Taggart L, Coates V, McAloon T, Hassiotis A. A systematic review of the methodological and practical challenges of undertaking randomised-controlled trials with cognitive disability populations. *Soc Sci Med* 2018;**200**:114-28.

20. Mitchell AJ, Selmes T. Why don't patients attend their appointments? Maintaining engagement with psychiatric services. *Adv Psychiatr Treat* 2007;**13**(6):423-34.

21. Deb S, Kwok H, Bertelli M, Salvador-Carulla L, Bradley E, Torr J, et al. International guide to prescribing psychotropic medication for the management of problem behaviours in adults with intellectual disabilities. *World Psychiatry* 2009;**8**(3):181-6.

22. Gibson RC, Bouamrane MM, Dunlop M. Design Requirements for a Digital Aid to Support Adults With Mild Learning Disabilities During Clinical Consultations: Qualitative Study With Experts. *JMIR Rehab* 2019;**6**(1):e10449.

23. Huxley CJ, Atherton H, Watkins JA, Griffiths F. Digital communication between clinician and patient and the impact on marginalised groups: a realist review in general practice. *Br J Gen Pract* 2015;**65**(641):e813-e821.

For peer review only

24. Sheehan R, Kimona K, Giles A, Cooper V, Hassiotis. Findings from an online survey of family carer experience of the management of challenging behaviour in people with intellectual disabilities, with a focus on the use of psychotropic medication. *Br J Learn Disabil* 2018;**46**(2):82-91.

25. Mehta H, Glover G. *Psychotropic drugs and people with learning disabilities or autism.* Public Health England, 2019.

26. Anderson K, Stowasser D, Freeman C, Scott I. Prescriber barriers and enablers to minimising potentially inappropriate medications in adults: a systematic review and thematic synthesis. *BMJ Open* 2014;**4**(12):e006544.

27. Reeve E, To J, Hendrix I, Shakib S, Roberts MS, Wiese MD. Patient barriers to and enablers of deprescribing: a systematic review. *Drugs Aging* 2013;**30**(10):793-807.

28. Ahmed Z, Fraser W, Kerr MP, Kiernan C, Emerson E, Robertson J, et al. Reducing antipsychotic medication in people with a learning disability. *Br J Psychiatry* 2000;**176**(1):42-6.

29. Shankar R, Wilcock M, Deb S, Goodey R, Corson E, Pretorius C, et al. A structured programme to withdraw antipsychotics among adults with intellectual disabilities: The Cornwall experience. *J Appl Res Intellect Disabilil* 2019; epub ahead of print.

30. Michie S, Van Stralen MM, West R. The behaviour change wheel: a new method for characterising and designing behaviour change interventions. *Implement Sci* 2011;**6**(1):42.

31. Zaal RJ, Ebbers S, Borms M, de Koning B, Mombarg E, Ooms P, et al. Medication review using a Systematic Tool to Reduce Inappropriate Prescribing (STRIP) in adults with an intellectual disability: A pilot study. *Res Dev Disabil* 2016;**55**:132-42.

32. Scheifes A, Egberts TC, Stolker JJ, Nijman HL, Heerdink ER. Structured medication review to improve pharmacotherapy in people with intellectual disability and behavioural problems. *J Appl Res Intellect Disabilil* 2016;**29**(4):346-55.

33. Bach LL, Lazzaretto DL, Young CF, Lofholm PW. Improving nursing home compliance via revised antipsychotic use survey tool. *Consult Pharm* 2017;**32**(4):228-38.

For peer review only - http://bmjopen.bmj.com/site/about/guidelines.xhtml

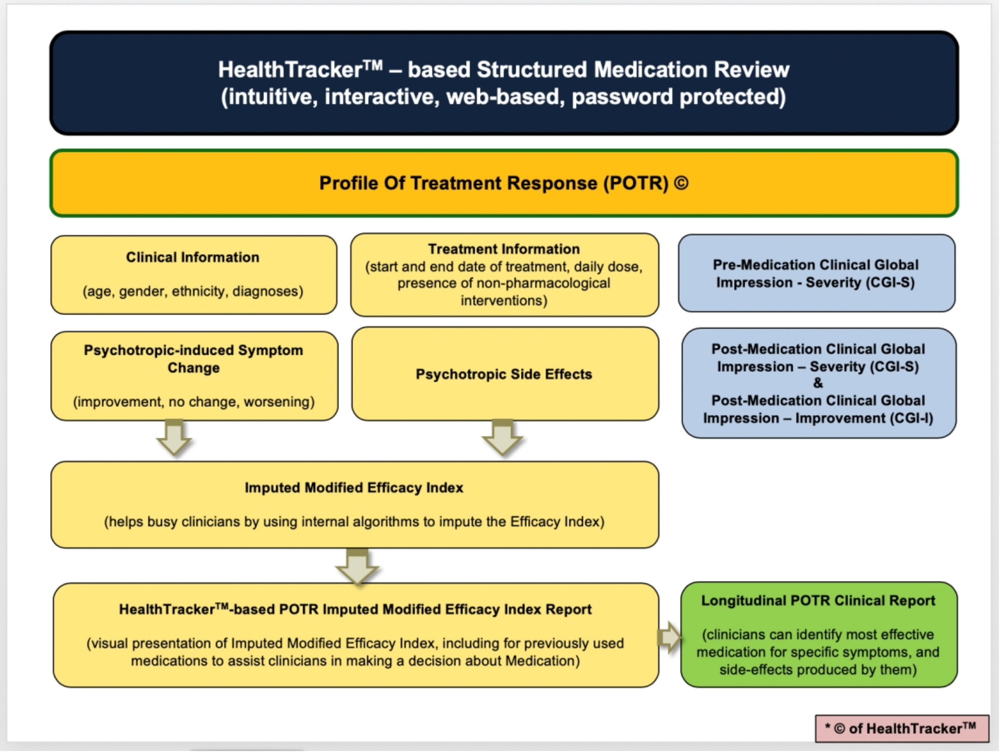

HealthTrackerTM-based structured medication review (HT-SMR)

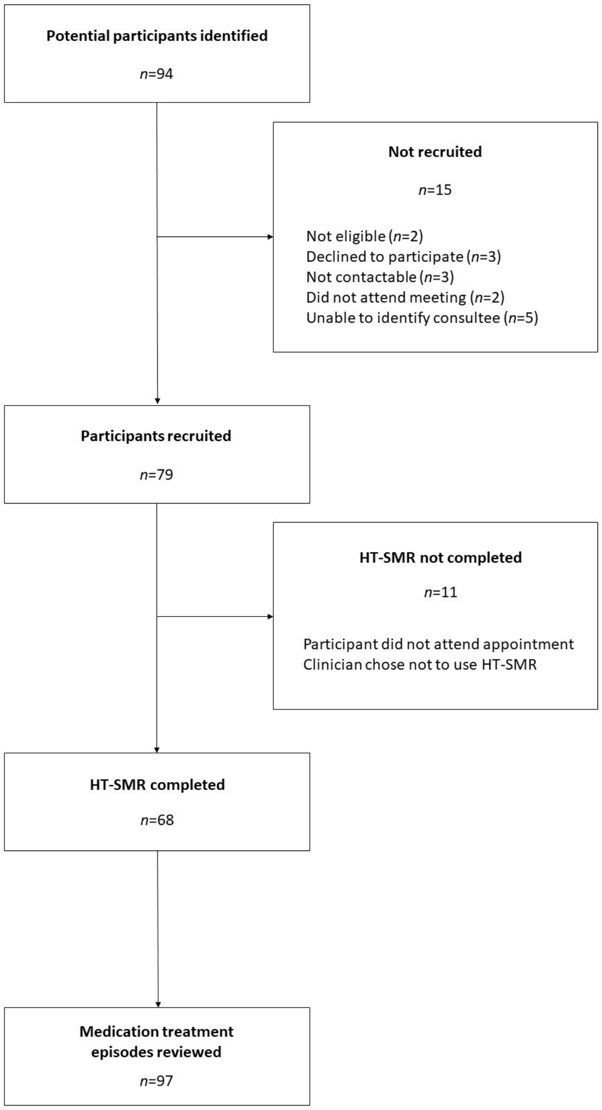

Participant flow

338x451mm (96 x 96 DPI)

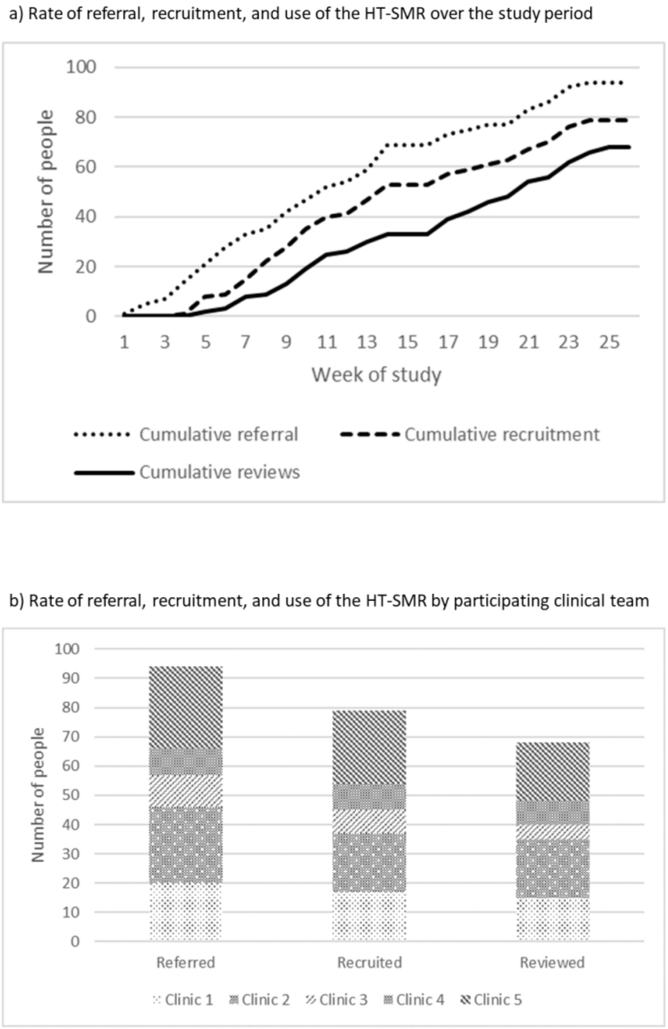

a) Rate of referral, recruitment, and use of the HT-SMR over the study period, and b) by participating clinical team

338x451mm (96 x 96 DPI)

BMJ Open

A structured medication review tool to promote psychotropic medication optimisation for adults with intellectual disability: feasibility study

Journal:	*BMJ Open*
Manuscript ID	bmjopen-2019-033827.R1
Article Type:	Original research
Date Submitted by the Author:	09-Oct-2019
Complete List of Authors:	Sheehan, Rory; University College London, Division of Psychiatry<br>Strydom, André; Institute of Psychiatry Psychology and Neuroscience<br>Marston, Louise; University College London, Research Department of Primary Care and Population Health<br>Morant, Nicola; University College London, Division of Psychiatry<br>Fiori, Federico; Institute of Psychiatry, Psychology & Neuroscience, Department of Child and Adolescent Psychiatry<br>Santosh, Paramala; King's College London , Department of Child and Adolescent Psychiatry<br>HASSIOTIS, ANGELA; University College London, Division of Psychiatry
**Primary Subject Heading**:	Mental health
Secondary Subject Heading:	Pharmacology and therapeutics
Keywords:	intellectual disability, psychotropic medication, medication optimisation, medication review, feasibility study

SCHOLARONE™
Manuscripts

TITLE PAGE

A structured medication review tool to promote psychotropic medication optimisation for adults with intellectual disability: feasibility study

Dr Rory Sheehan, MSc, Division of Psychiatry, University College London, London, UK

Professor André Strydom, PhD, Institute of Psychiatry, Psychology and Neuroscience, King's College London, London, UK

Dr Louise Marston, PhD, Research Department of Primary Care and Population Health, Division of Psychiatry, University College London, London, UK

Dr Nicola Morant, PhD, Division of Psychiatry, University College London, London, UK

Dr Federico Fiori, Institute of Psychiatry, Psychology and Neuroscience, King's College London, London, UK

Professor Paramala Santosh, Institute of Psychiatry, Psychology and Neuroscience, King's College London, London, UK

Professor Angela Hassiotis, PhD, Division of Psychiatry, University College London, London, UK

Corresponding author details

Dr Rory Sheehan

Division of Psychiatry, University College London, 6th Floor Maple House, 149 Tottenham Court Road, London W1T 7NF

Telephone: 0207-679-9201

E-mail: r.sheehan@ucl.ac.uk

For peer review only - http://bmjopen.bmj.com/site/about/guidelines.xhtml

Word count

4,130

Number of figures and tables

2 tables

3 figures

Keywords

Intellectual disability; psychotropic medication; medication optimisation; medication review; feasibility study

For peer review only

A structured medication review tool to promote psychotropic medication optimisation for adults with intellectual disability: feasibility study

ABSTRACT

Objectives To investigate the feasibility of delivering structured psychotropic medication review in community services for adults with intellectual disability.

Design Single-arm feasibility study conducted over a six-month period.

Setting Specialist community intellectual disability teams in England.

Participants Psychiatrists working with adults with intellectual disability and adults with intellectual disability who had been prescribed psychotropic medication.

Intervention A structured web-based psychotropic medication review tool (the HealthTracker^{TM}-based structured medication review) comprising measures of therapeutic benefit and adverse side-effects was made available for use by psychiatrists in routine clinic appointments. A summary measure of medication effectiveness was graphically presented to aid discussion and decision-making.

Main outcome measures Feasibility metrics including number of people with intellectual disability referred, eligible, and recruited, and uptake of the medication review tool in

For peer review only - http://bmjopen.bmj.com/site/about/guidelines.xhtml

naturalistic clinical settings. Psychiatrist and patient feedback was collected to assess acceptability of the intervention and suggestions for development.

Results Fifteen psychiatrists from five clinical teams took part. In total 94 potentially-eligible people with intellectual disability were referred, of whom 79 (84%) were recruited and together underwent 97 medication reviews over the six month study period. Feedback from participants with intellectual disability was favourable. Psychiatrists indicated the HealthTracker™-based medication review was broadly acceptable and suggested adaptations to improve integration with existing information technology systems and to enhance patient involvement in the review.

Conclusions Structured psychotropic medication review can be used in community services for adults with intellectual disability as part of a programme of medication optimisation. It would be feasible to test clinical and patient outcomes of the HealthTracker™-based medication review in a randomised clinical trial.

For peer review only - http://bmjopen.bmj.com/site/about/guidelines.xhtml

STRENGTHS AND LIMITATIONS OF THIS STUDY

- This feasibility study is one of the first to suggest a pragmatic and scalable means of achieving psychotropic medication optimisation in people with intellectual disability using structured medication review.
- The work provides estimates of recruitment rate and uptake of the intervention, as well as suggestions for its development, that can inform the planning and delivery of a future clinical trial.
- The study was conducted in a single region of the UK which may not be representative of other locations or healthcare settings.
- Details of those who were eligible but did not participate in the study were not collected and the overall rate of uptake of the intervention cannot be determined.

For peer review only

INTRODUCTION

Intellectual disability (ID), present in approximately 2% of the population, is a lifelong disorder defined by significant cognitive deficit and impaired functional and adaptive skills.[1] Between a third and one half of adults with ID are prescribed psychotropic medication.[2, 3] Renewed focus on the quality of prescribing has been prompted by epidemiological evidence which shows that the extent of psychotropic use is disproportionate to prevalence of mental illness in this group, and medication is often used ‘off-label’ in the management of behaviour that challenges.[4] People with ID are at greater risk of idiosyncratic reactions and adverse medication side-effects than their non-intellectually disabled counterparts and are more likely to receive high psychotropic doses, polypharmacy, and to remain on psychotropic medication for extended periods.[5, 6]

The UK Government has committed to improving the use of psychotropic medication in people with ID[7] and a national programme, Stopping the Over-Medication of People with Learning Disabilities (STOMP), was established in 2016 to raise awareness of the issue and stimulate activity amongst patients, advocates, and professionals.[8] Medication optimisation is a multi-faceted concept that aims to promote the best use of prescribed medication by prioritising safety, evidence-based choice of medication, and centring patient experience and involvement.[9] Medication review, a structured and critical evaluation of a prescribed medication, is a key element of medication optimisation that is recommended by the National Institute for Health and Care Excellence (NICE) for groups at high risk of suboptimal medication use.[10] Structured medication review offers a number of potential benefits including: promoting systematic evaluation of desired and undesired medication effects;

For peer review only - http://bmjopen.bmj.com/site/about/guidelines.xhtml

standardisation of assessment across time and between clinicians; an efficient method of recording information; and making explicit the basis on which decisions are made. A recent systematic review found that psychotropic medication review is associated with change or reduction in number of drugs prescribed but consistent improvement in clinical and patient-reported outcomes has not been shown, and there is considerable variation and little formal guidance on how medication reviews are operationalised.[11] We undertook a study to investigate the feasibility of a structured psychotropic medication review (the HealthTracker™-based structured medication review, HT-SMR) in community psychiatry of ID teams. Specific objectives were to determine how recruitment of psychiatrists and people with ID to the study, to assess the uptake of this novel intervention in real-world clinical settings, and to gather feedback that could inform future development and refinement of the intervention.

METHOD

Study procedures

This was a single-arm feasibility study conducted over a six-month period in five community psychiatry of ID services in London, UK. All services were part of the National Health Service. The study and its rationale were presented to psychiatrists in participating clinical teams, and they were then invited to take part in the study. If they agreed, they were given access to the HT-SMR for the study period. Adults (>18 years) with ID were eligible to participate if they were prescribed psychotropic medication of any type and for any indication.

For peer review only - http://bmjopen.bmj.com/site/about/guidelines.xhtml

Psychiatrists were asked to briefly introduce the research to potential participants and/or their carers, either at routine appointments or by sending an information leaflet through the post. The contact details of those who expressed interest were passed to the research team who then met with the potential participant to explain the research in more detail and confirm eligibility. Written informed consent was obtained from all people with ID. Ability to consent to take part was assessed according to the principles of the Mental Capacity Act.[12] If a person lacked capacity to consent, a family member or nominated consultee was sought to give advice to the research team on the person's inclusion. All study materials were available in accessible (easy-read) format. When a participant was recruited to the study, their psychiatrist was informed and was then able to use the HT-SMR in appointments with that person.

Intervention

The intervention consisted of the HT-SMR (figure 1) designed to be used in routine clinical appointments by a participant's psychiatrist. The HealthTracker™ is a password-protected web-based health monitoring platform that originated in the NHS, with the NHS receiving royalties from its use. For the purposes of this study, medication review included a record of basic demographic, clinical and treatment information along with responses to the Profile of Treatment Response (POTR). The POTR comprises two generic scales; one measuring therapeutic response to a medication over several symptom domains, the other measuring potential adverse side-effects. Each item is rated by the psychiatrist on a Likert scale using information gathered from observation and the clinical interview. Items that are not applicable can be marked as such but incomplete reviews cannot be submitted. Based on

For peer review only - http://bmjopen.bmj.com/site/about/guidelines.xhtml

responses to the two scales above, the HealthTracker™ imputes the Modified Efficacy Index (MEI) as the ratio between the therapeutic benefit of a medication and the presence of adverse side-effects. The MEI is then displayed in a simple colour-coded matrix that allows viewers to see how the patient has responded to treatment and may act as a stimulus for discussion between the psychiatrist and the patient and/or carer. The Clinical Global Impression-Improvement (CGI-I), a well-established rating tool that can be completed quickly and easily in clinical settings,[13] is completed by the psychiatrist for each medication as a further measure of medication effect. We asked psychiatrist to record if they had advised a change to medication following the review.

FIGURE 1 NEAR HERE - HealthTracker™-based structured medication review (HT-SMR)

Each participant with ID was assigned a unique identification number and pseudonymised data collected in the medication review were stored on a secure electronic cloud. A single medication or multiple medications could be reviewed at one time, with a separate POTR and separate CGI-I for each drug that was reviewed. If the HT-SMR is used across different time periods, a longitudinal record of treatment response to a certain medication is generated. The researcher trained psychiatrists on using the system in face-to-face small-group sessions focused on the practicalities of opening a case and entering data, and used a fictional patient to reinforce the learning. The research team were available throughout the study for support as needed.

For peer review only - http://bmjopen.bmj.com/site/about/guidelines.xhtml

Data from medication reviews were downloaded from the HealthTracker™ as a CSV file into SPSS v.24 at the end of the study period. The POTR, MEI and CGI-I results were summarised with descriptive statistics. Spearman's correlation between the MEI and CGI-I and the psychiatrist's decision to change or not change medication were calculated. Owing to the skewness of the data, non-parametric tests were used to test the significance of associations.

Feasibility measures

We gauged interest from clinical teams and individual psychiatrists to take part in the study and recorded the rates of referral and recruitment of people with ID, and of uptake of the medication review tool in routine clinic appointments. Reasons for not recruiting people who were referred to the research team were noted. As this was a feasibility study, a formal sample size calculation was not performed but our *a priori* estimate was that 100 people with ID would be recruited, based on previous feasibility studies that have trialled similar interventions in community settings.[14]

Participant characteristics

Characteristics of people with ID who were recruited and descriptive data concerning diagnosis and medication use are reported. Medication doses were converted to defined daily dose (DDD).[15]

Acceptability and implementation

For peer review only - http://bmjopen.bmj.com/site/about/guidelines.xhtml

For peer review only

At the end of each medication review, psychiatrists asked people with ID, "How able were you to say everything you wanted to say about medication today?" Answers were scored on a five-point Likert scale with pictorial cues alongside the response set to improve understanding. At the end of the study period, psychiatrists were invited to complete an anonymous web-based survey designed for this study with a mix of closed and open-ended questions. The survey concerned the research process, experience and views on use of the online review system, and suggested adaptations to maximise usability and utility of the medication review in its future development. Responses to the psychiatrist feedback questionnaire were summarised in a structured analysis within pre-determined categories. All data were managed in SPSS v.24 and Microsoft Excel.

Patient and public involvement

A service user consultation group was formed as part of the wider programme of work within which this study was conducted. The consultation group consisted of people with ID and experience of medication use. We held regular meetings with the consultation group, who advised on various aspects of this work including, the recruitment strategy, participant materials and easy-read information, devising the outcome measure for participants with ID, and general advice to aid successful conduct of the research. The group will be involved in dissemination to a broad range of relevant stakeholders.

Ethical approval

For peer review only - http://bmjopen.bmj.com/site/about/guidelines.xhtml

The authors assert that all procedures contributing to this work comply with the ethical standards of the relevant national and institutional committees on human experimentation and with the Helsinki Declaration of 1975, as revised in 2008. All procedures involving patients were approved by the London Bridge Research Ethics Committee (ref: 18/LO/1112).

RESULTS

Recruitment and uptake

Five community ID teams comprising fifteen psychiatrists were invited and agreed to take part in the feasibility study which was conducted between September 2018 and March 2019. Eight psychiatrists were of consultant grade (who had completed specialist training in psychiatry of ID) and seven psychiatrists were trainees (with between 6 months and 3 years' experience working in with people with ID). Together, 94 people with ID were referred as potential participants over the six-month study period and 79 (84%) were recruited. Psychiatrists used the online system for medication review in 68 people (86% of those recruited). A number of people (*n*=21) had more than one medication review (when either more than one medication was reviewed at a single time point, or a single medication was reviewed on more than one occasion) giving a total of 97 HT-SMRs (figure 2).

FIGURE 2 NEAR HERE - Participant flow

For peer review only - http://bmjopen.bmj.com/site/about/guidelines.xhtml

There was a steady state of referral, recruitment and review tool use (figure 3a). Recruitment and uptake of the HT-SMR was unequal between participating community ID teams and not related to the number of psychiatrists in each of these teams (figure 3b). Each psychiatrist conducted a median of 7 medication reviews using the HT-SMR (range 0-20). No harms or unintended consequences were reported during the study and no participants withdrew their consent.

FIGURE 3 NEAR HERE – a) Rate of referral, recruitment, and use of the HT-SMR over the study period, and b) by participating clinical team

Participant information and data from medication reviews

Demographic data of participants with ID who had medication review are summarised in table 1. The group was relatively young and most had mild ID. A primary diagnosis was not recorded in just over half of the participants; in these cases it is possible that psychotropic medication was prescribed for behaviour that challenges.

For peer review only

Table 1 Demographic characteristics of participants with ID

**Characteristic**	***n* (%)**
**Sex**	
Male	41 (60%)
Female	27 (40%)
**Age at first HT-SMR (years)**	
18-25	23 (34%)
26-35	16 (24%)

For peer review only - http://bmjopen.bmj.com/site/about/guidelines.xhtml

36-45	8 (12%)
46-55	17 (25%)
55-65	1 (1%)
>65	3 (4%)
**Degree of ID**	
Mild	42 (62%)
Moderate	18 (26%)
Severe-profound	8 (12%)
**Ethnicity**	
White	35 (51%)
Black	14 (21%)
Asian	10 (15%)
Mixed / other	7 (10%)
Not known / not given	2 (3%)
**Primary diagnosis**	
Schizophrenia spectrum disorder	12 (18%)
Mood disorder	5 (7%)
Anxiety disorder	3 (4%)
Personality disorder	1 (1%)
Pervasive developmental disorder	7 (10%)
Attention deficit hyperactivity disorder	3 (4%)
Missing	37 (54%)

For peer review only

Of the 97 HT-SMRs conducted using the system, the most commonly reviewed drug class was antipsychotics (49 reviews), followed by anti-depressants (28 reviews) (table 2). The median prescribed dose of medication reviewed was 100% DDD (inter-quartile range, IQR, 50-133%) and median duration of use was 18 months (IQR, 5-56 months). Following the HT-SMR, psychiatrists advised a change to medication in just over one-third (*n*=27, 36%) cases.

For peer review only - http://bmjopen.bmj.com/site/about/guidelines.xhtml

Table 2 Summary results from the HT-SMRs (*n*=97 reviews)

Drug class reviewed	Number of reviews (% of all reviews)	Median DDD of medication reviewed (IQR)	Median duration of use (months) (IQR)	Median CGI-Improvement (IQR)*	Median Modified Efficacy Index (IQR)†
**Antipsychotic**	49 (51%)	67 (45-100)	24 (4-60)	1.5 (1.0-2.0)	2.0 (1.0-3.0)
**Anti-depressant**	28 (29%)	150 (87-200)	12 (4-24)	2.0 (1.0-3.0)	2.0 (1.4-3.0)
**Anxiolytic / sedative**	9 (9%)	24 (2-54)	100 (39-100)	3.0 (2.0-3.0)	3.0 (2.3-4.0)
**Medication for ADHD**	9 (9%)	120 (78-138)	18 (12-78)	2.0 (1.0-2.5)	2.0 (1.0-4.0)
**Mood stabiliser**	2 (2%)	33	96	2.5	1.2
**All**	97 (100%)	100 (50-133)	18 (5-56)	2.0 (1.0-3.0)	1.5 (1.0-3.0)

IQR, inter-quartile range

*CGI is scored between 1 (very much improved) and 7 (very much worse)

†Modified Efficacy Index is the ratio between the score in the domain with the greatest therapeutic benefit to the score in the domain with the worst rated adverse side-effect. Higher scores indicate more favourable medication response.

The HealthTracker^{TM} imputed MEI can take a value of 0.33 to 4.0, where higher values equate to a more favourable therapeutic effect:adverse side-effect ratio. The median HealthTracker^{TM} imputed MEI for medications reviewed was 1.5 (IQR 1.0-3.0). There was a

For peer review only - http://bmjopen.bmj.com/site/about/guidelines.xhtml

statistically-significant negative correlation between the MEI and the CGI-I (where a lower score indicates greater perceived benefit of medication) (ρ -0.296, *p*=0.024; indicating 'fair' correlation between the two measures).[16] The MEI was significantly lower in those in whom a medication change made following the review (median MEI 1.0, IQR 0.67-2.0) compared with those in whom no medication change was made following the review (median MEI 1.5, IQR 1.3-3.0) (*p*=0.011).

Acceptability and implementation

When asked "How able were you to say everything you wanted to say about medication today?", participants with ID responded "very easy" or "easy" in 54 (70%) cases, "not easy or difficult" in 14 (18%) cases, "difficult" or "very difficult" in 1 (1%) case. The question was not answered by 9 (12%) participants with ID.

Fourteen psychiatrists out of fifteen completed the online feedback questionnaire. Results are presented as major themes with anonymised quotations to illustrate points of interest.

Feedback about the recruitment process

Although the majority (13/14) of psychiatrists reported that it had been 'easy' to introduce the study to potential participants, most (11/14) had encountered barriers. The main barriers to recruitment were *"time constraints"* within appointments and difficulties explaining the research to potential participants, especially those with more severe ID.

For peer review only - http://bmjopen.bmj.com/site/about/guidelines.xhtml

Psychiatrists were asked if the people who did not wish to hear more about the research had given reasons for their decision. The most commonly reported reason (8 cases) was worry about the commitment or inconvenience the research would entail. Others declined to hear more as they were already taking part in research or were content with their current medication regimen and did not want to discuss this further. Seven psychiatrists reported that the person's carer had not wished to pursue the research opportunity, either because they felt it was not appropriate or because they were not willing to act as a consultee in cases where the person with ID was likely to lack capacity to provide informed consent.

Ease of using the HealthTracker™ online system

Twelve psychiatrists reported having used the online system for medication review. In response to the question, "How easy was it to use the HealthTracker?" only one person reported it was "difficult"; the majority said it was "easy" or "very easy".

Benefits of HT-SMR

Eight out of 12 psychiatrists were of the opinion that using the online medication review had helped people with ID or their carer to be more involved in the discussion about medication and promoted *"collaborative decision-making"*. Psychiatrists commented that it had been *"helpful as a template"* in *"framing the discussion around medication"* and that its use facilitated *"more in-depth"* and *"comprehensive"* medication review. The transparency of medication review using the system was described as an advantage: *"[using the tool] was an eye-opener for the patient and carer. They could hear the specific questions being asked*

For peer review only - http://bmjopen.bmj.com/site/about/guidelines.xhtml

systematically and seeing the matrix [the graphical representation of the EI] was really useful, particularly for a few participants who were not clear about medication".

Disadvantages of the HT-SMR

Six psychiatrists described logistical problems in using a system which required internet connectivity e.g. computers not working or running fast enough, lack of internet access in clinic settings, not having portable devices for domiciliary visits. Using the system took additional time which was sometimes difficult to find in the regular appointment.

Just over half (7/12) of psychiatrists expressed the view that using the online medication review had interfered with their interaction with the patient or carer with one remarking that they had spent *"more time focussed on the computer rather than face-to-face personal interaction"*. Two psychiatrists considered the system too rigid and resisted the *"imposed structure"* of the medication review which they believed was not always aligned with the patient's most pressing concerns.

Effect on decision-making

Eight out of 12 psychiatrists thought that undertaking the HT-SMR had helped them to make a decision about medication and 5/12 considered the tool made it more likely they would change medication compared with their usual practice. However in the survey free-text responses most commented that the medication review did not cause them to change decisions they would ordinarily have made, rather, the HT-SMR was viewed as *"an*

For peer review only - http://bmjopen.bmj.com/site/about/guidelines.xhtml

additional tool" which could *"confirm a clinical impression," "justify decisions"* and give clinicians *"more confidence"*.

Adaptations and views about future use

Eight out of 12 psychiatrists thought that SMR should be used more widely. Suggestions to improve the system centred on making the system more *"user friendly"* and *"intuitive"* for psychiatrists, and integrated with existing computerised systems. Three psychiatrists also mentioned improving the accessibility to people with ID incorporating their views more formally in the medication review e.g. *"adding a weight to the [decision-support] algorithm based on patient preference"*.

DISCUSSION

Main findings

There is a need to improve the quality of psychotropic medication use in people with ID yet despite consensus guidelines of good practice,[17, 18] there has been relatively little work to investigate practical methods to achieve medication optimisation in this group. The current study introduced a structured medication review tool in community psychiatric services for adults with intellectual disability and demonstrates that it would be feasible to test outcomes in a definitive trial.

For peer review only - http://bmjopen.bmj.com/site/about/guidelines.xhtml

Perhaps owing to the scrutiny currently applied to psychotropic prescribing, clinical teams and psychiatrists that we approached were keen to take part in this research. Recruitment of people with ID to research can be challenging[19] but the number of participants we recruited was satisfactory, close to our original broad expectation, and the referral:recruitment ratio was high, indicating the processes of participant identification, recruitment and consent were appropriate.

A key question was whether psychiatrists were able and willing to integrate use of the HT-SMR into their standard practice, given the demands on their time and numerous mandated clinical and administrative tasks. Uptake of the HT-SMR was good, though not universal; three psychiatrists did not use the tool and eleven people with ID who were recruited did not have a recorded medication review. The rate of missed appointments is higher in psychiatric clinics than in other medical specialties[20] and may be higher still in ID services and missed appointments are one likely cause that limited the HT-SMR during the study period.

The HealthTracker™ imputed MEI was tested as a potential future outcome measure. The MEI was correlated with the overall CGI-I and was lower (indicating a less favourable risk:benefit ratio) in those in whom medication changes were made compared with those in whom medication remained unchanged. The HealthTracker™ imputed MEI showed sufficient variation between participants and had value as a practical support to psychiatrists in considering medication changes, though the survey data showed that this does not replace psychiatrists' clinical judgement. However, there may also be disadvantages to using a single measure of medication effect in those who receive

For peer review only - http://bmjopen.bmj.com/site/about/guidelines.xhtml

polypharmacy as psychiatrists (and patients and their carers) may find it difficult to attribute changes to a specific medication.

This research involved relatively little commitment from participants with ID and the intervention appeared acceptable in view of the recruitment metrics and response to the evaluation questionnaire. Two-thirds of psychiatrists thought the system should be used more extensively, indicating an overall favourable attitude. In order to maintain proximity to usual practice, we gave psychiatrists flexibility and few instructions of how to use the online system in their appointments, other than on how to enter data. There was clearly variation in how different psychiatrists approached the HT-SMR; positive feedback showed that some appreciated the systematic and comprehensive nature of the medication review and believed that it could facilitate a discussion with the person with ID. Negative comments referred to the perception that the structured review was inflexible and rigid. This may be related to natural variation in clinicians' consultation style and familiarity with incorporating standardised or structured elements to the consultation, although these are recommended in monitoring medication effects.[18, 21]

Some psychiatrists reported disruption to the relational aspects of the consultation arising from the need to interact simultaneously with the computer screen and the person with ID and others who may attend the appointment. Electronic records are already used extensively in healthcare settings but use of technology as a more dynamic application may represent a more profound culture change and requires the development of new skills and ways of working. It is possible that digital interventions, if properly designed, can enhance communication between doctor and patient, for example, by incorporating augmentative

For peer review only - http://bmjopen.bmj.com/site/about/guidelines.xhtml

and alternative communication methods.[22, 23] Given that patient involvement and the opportunity for shared decision-making is fundamental to medication optimisation, the HT-SMR would benefit from incorporating a greater role for people with ID and their carers to amplify the patient voice. This could go some way to countering the lack of involvement that patients and their carers often describe when medication decisions are made.[24] Other opportunities to extend the remit of this system include patients or carers completing measures in advance of appointments in order to release consultation time for discussion and collaboration, particularly if the system were configured to prioritise the individuals' indication for medication and the most common adverse side-effects of the drug prescribed.

Future work

A future clinical trial is needed to test if use of the HT-SMR contributes to medication optimisation. The HealthTracker™ imputed MEI could be used as a primary outcome measure and should be supplemented by other measures of medication optimisation, including service utilisation, medication safety incidents, and patient-reported outcomes, including decision self-efficacy and satisfaction. An economic evaluation is also necessary to determine the cost implications of the intervention; balanced against the additional resource and infrastructure necessary to deliver the HT-SMR are potential cost savings achieved through reductions in medication waste and in indirect costs related to adverse side-effects.

Widescale implementation of a system of structured medication review would create a powerful naturalistic dataset of medication use, therapeutic impact, and adverse side-

For peer review only - http://bmjopen.bmj.com/site/about/guidelines.xhtml

effects that could be used both as a dashboard to monitor and benchmark prescribing practice, and for observational research in this group where there is a paucity of empirical data and little prospect of significant future controlled trials.

The STOMP campaign in England has so far not achieved discernible reductions in psychotropic prescribing to adults with ID.[25] Medication review, as an opportunity for critical reflection and discussion about medication, may act as a stimulus for change in prescribing that will ultimately improve medication outcomes. However, there are many influences on prescribing behaviour, including those acting on an individual level amongst patients, carers and clinicians,[26, 27] as well as systemic factors which are likely to extend beyond the control of the prescriber, such as appropriately-supported accommodation and social care provision.[28] Thus, a medication review intervention can only be one element of a programme of medication optimisation and changing behaviour on a wider scale will require concerted action across health and social care sectors. One published report of a multi-component intervention to reduce antipsychotic use has shown some success but was time-consuming, has not been replicated, and lacks longer-term outcomes.[29] Future evidence-based complex interventions (of which structured medication review can be a part) that can work at scale should be underpinned by a theoretical framework that can identify the levers and barriers that are most likely to affect implementation.[30]

Strengths and limitations of this study

This study was completed in real-world settings, included psychiatrists of different grades from several different services, and a diverse group of participants, thereby increasing

For peer review only - http://bmjopen.bmj.com/site/about/guidelines.xhtml

generalisability of the findings. We obtained estimates of important recruitment parameters and confirmed a successful recruitment strategy. Feedback has enabled us to identify aspects of the HT-SMR which require development to improve utility and enhance the potential for benefits of the intervention. The advantages of this mediation review were that it is relatively quick, self-explanatory, and can be completed in a single patient contact, making it easier to integrate into the current models of care than other published medication review methods that are multi-stage and multi-professional and more likely to encounter implementation barriers.[31, 32] Being conducted by the psychiatrist, who is also the prescriber, avoids the pitfalls of non-prescriber directed medication reviews in which as few as one-third of recommendations are actioned.[33]

This study also had limitations. We could not collect the characteristics of those who declined to participate in the research, and therefore do not know the total eligible population or whether certain groups were under-represented in our sample. Similarly, we do not know the number of appointments in which the system could have been used in but was not, and without this denominator cannot report the rate of uptake. Attrition and clinician fatigue in using the online medication review may be an issue in a longitudinal study that was not addressed in this feasibility study, given the relatively short time-period of the research. A single participant feedback question was chosen to minimise demands placed on participants but was inevitably limited in scope and responses may have been subject to social desirability bias. Although logic suggests that the medication review would give patients and carers a greater opportunity for input in the process of medication decision-making, this was not formally tested and there was no method for gathering feedback from carers who may have been involved in the appointment and who play an

For peer review only - http://bmjopen.bmj.com/site/about/guidelines.xhtml

important role in the medication process. We also included only a limited number of psychiatrists, and within this group some were more enthusiastic users of the HT-SMR than others. This introduces a further source of bias, as the results are largely driven by only a small number of psychiatrist users of the system.

Conclusion

Medication review has potential to improve individual medication outcomes as part of a wider programme of medication optimisation. The HT-SMR could be tested in a definitive trial after some refinement to improve integration with existing software and to fully embed patient and carer voice in the review process.

For peer review only

For peer review only - http://bmjopen.bmj.com/site/about/guidelines.xhtml

Acknowledgements

The authors would like to thank staff in the clinical services who recruited participants and completed the HT-SMR, members of the Camden Synergy service user consultation group (Jill Huntesmith and Jackie McMorrow), and the research participants and their carers.

Author contribution statement

RS, AS, LM, NM, FF, PS, and AH conceived and designed the study and contributed towards the conduct and management of the work. RS recruited participants. RS, FF, and LM managed and analysed the data and AS, NM, PS, and AH supervised this process. RS, AS, LM, NM, FF, PS, and AH interpreted the results. RS wrote the first draft of the paper and AS, LM, NM, FF, PS, and AH contributed to further drafts and read and approved the final manuscript.

Funding

RS is supported by a Doctoral Research Fellowship from the National Institute for Health Research (NIHR) (Ref: DRF-2016-09-140).

Disclaimer

For peer review only - http://bmjopen.bmj.com/site/about/guidelines.xhtml

For peer review only

The views expressed in this article are those of the authors and not necessarily those of the NIHR or the Department of Health and Social Care. The funder had no role in study design, analysis, decision to publish, or preparation of the manuscript.

Data sharing

No additional data are available.

Competing interests

All authors have read and completed the ICMJE form for competing interests.

Dr Sheehan reports no conflict of interest.

Professor Strydom reports no conflict of interest.

Dr Marston reports no conflict of interest.

Dr Morant reports no conflict of interest.

Dr Fiori reports that he is employed as Chief Technology Officer by HealthTracker Ltd - the company that has the copyright for the HealthTracker™-based Structured Medication Review (HT-SMR). The HT-SMR can be licensed to hospitals and healthcare settings.

Professor Santosh reports that he is a Director and shareholder of HealthTracker Ltd - the company that has the copyright for the HealthTracker™-based Structured Medication Review (HT-SMR). The HT-SMR can be licensed to hospitals and healthcare settings.

Professor Hassiotis reports no conflict of interest.

For peer review only

REFERENCES

1. American Psychiatric Association, editors. *Diagnostic and statistical manual of mental disorders: DSM-5)*: American Psychiatric Association, 2013.

2. Tsiouris JA, Kim S-Y, Brown WT, Pettinger J, Cohen IL. Prevalence of psychotropic drug use in adults with intellectual disability: positive and negative findings from a large scale study. *J Autism Dev Disord* 2013;**43**(3):719-31.

3. Bowring D, Totsika V, Hastings R, Toogood S, McMahon M. Prevalence of psychotropic medication use and association with challenging behaviour in adults with an intellectual disability. A total population study. *J Intellect Disabil Res* 2017;**61**(6):604-17.

4. Sheehan R, Hassiotis A, Walters K, Osborn D, Strydom A, Horsfall L. Mental illness, challenging behaviour, and psychotropic drug prescribing in people with intellectual disability: UK population based cohort study. *BMJ* 2015;**351**:h4326.

5. Straetmans JM, van Schrojenstein Lantman-de HM, Schellevis FG, Dinant G-J. Health problems of people with intellectual disabilities: the impact for general practice. *Br J Gen Pract* 2007;**57**(534):64-6.

6. De Kuijper G, Hoekstra P, Visser F, Scholte F, Penning C, Evenhuis H. Use of antipsychotic drugs in individuals with intellectual disability (ID) in the Netherlands: prevalence and reasons for prescription. *J Intellect Disabil Res* 2010;**54**(7):659-67.

7. NHS England. *NHS Long Term Plan.* NHS England, 2019 (https://www.longtermplan.nhs.uk)

8. Branford D, Gerrard D, Saleem N, Shaw C, Webster A. Stopping over-medication of people with an intellectual disability, autism or both (STOMP) in England part 2–the story so far. *Advances in Mental Health Intellectual Disabilities* 2019;**13**(1):41-51.

9. Royal Pharmaceutical Society. *Medicines Optimisation: helping patients to make the most of medicines.* Royal Pharmaceutical Society, 2013.

10. National Institute for Health and Care Excellence. *Medicines optimisation: the safe and effective use of medicines to enable the best possible outcomes. NICE Guideline NG5.* National Institute for Health and Care Excellence, 2015.

For peer review only - http://bmjopen.bmj.com/site/about/guidelines.xhtml

For peer review only

11. Sheehan R, Strydom A, Brown E, Marston L, Hassiotis A. Association of focused medication review with optimization of psychotropic drug prescribing: a systematic review and meta-analysis. *JAMA Network Open* 2018;**1**(6):e183750.

12. Mental Capacity Act 2005. The Stationery Office, 2005.

13. Busner J, Targum SD. The clinical global impressions scale: applying a research tool in clinical practice. *Psychiatry (Edgmont)* 2007;**4**(7):28.

14. Moncrieff J, Azam K, Johnson S, Marston L, Morant N, Darton K, et al. Results of a pilot cluster randomised trial of the use of a Medication Review Tool for people taking antipsychotic medication. *BMC Psychiatry* 2016;**16**(1):205.

15. World Health Organization. ATC/DDD Toolkit. World Health Organisation (https://www.who.int/medicines/regulation/medicines-safety/toolkit_ddd/en/)

16. Chan Y. Biostatistics 104: correlational analysis. *Singapore Med J* 2003;**44**(12):614-9.

17. Shankar R, Wilcock M, Oak K, McGowan P, Sheehan R. Stopping, rationalising or optimising antipsychotic drug treatment in people with intellectual disability and/or autism. *Drug Ther Bull* 2019;**57**(1):10-3.

18. Royal College of Psychiatrists Faculty of Psychiatry of Intellectual Disability. *Psychotropic drug prescribing for people with intellectual disability, mental health problems and/or behaviours that challenge: practice guidelines.* Royal College of Psychiatrists, 2016.

19. Mulhall P, Taggart L, Coates V, McAloon T, Hassiotis A. A systematic review of the methodological and practical challenges of undertaking randomised-controlled trials with cognitive disability populations. *Soc Sci Med* 2018;**200**:114-28.

20. Mitchell AJ, Selmes T. Why don't patients attend their appointments? Maintaining engagement with psychiatric services. *Adv Psychiatr Treat* 2007;**13**(6):423-34.

21. Deb S, Kwok H, Bertelli M, Salvador-Carulla L, Bradley E, Torr J, et al. International guide to prescribing psychotropic medication for the management of problem behaviours in adults with intellectual disabilities. *World Psychiatry* 2009;**8**(3):181-6.

22. Gibson RC, Bouamrane MM, Dunlop M. Design Requirements for a Digital Aid to Support Adults With Mild Learning Disabilities During Clinical Consultations: Qualitative Study With Experts. *JMIR Rehab* 2019;**6**(1):e10449.

23. Huxley CJ, Atherton H, Watkins JA, Griffiths F. Digital communication between clinician and patient and the impact on marginalised groups: a realist review in general practice. *Br J Gen Pract* 2015;**65**(641):e813-e821.

For peer review only - http://bmjopen.bmj.com/site/about/guidelines.xhtml

For peer review only

24. Sheehan R, Kimona K, Giles A, Cooper V, Hassiotis A. Findings from an online survey of family carer experience of the management of challenging behaviour in people with intellectual disabilities, with a focus on the use of psychotropic medication. *Br J Learn Disabil* 2018;**46**(2):82-91.

25. Mehta H, Glover G. *Psychotropic drugs and people with learning disabilities or autism.* Public Health England, 2019.

26. Anderson K, Stowasser D, Freeman C, Scott I. Prescriber barriers and enablers to minimising potentially inappropriate medications in adults: a systematic review and thematic synthesis. *BMJ Open* 2014;**4**(12):e006544.

27. Reeve E, To J, Hendrix I, Shakib S, Roberts MS, Wiese MD. Patient barriers to and enablers of deprescribing: a systematic review. *Drugs Aging* 2013;**30**(10):793-807.

28. Ahmed Z, Fraser W, Kerr MP, Kiernan C, Emerson E, Robertson J, et al. Reducing antipsychotic medication in people with a learning disability. *Br J Psychiatry* 2000;**176**(1):42-6.

29. Shankar R, Wilcock M, Deb S, Goodey R, Corson E, Pretorius C, et al. A structured programme to withdraw antipsychotics among adults with intellectual disabilities: The Cornwall experience. *J Appl Res Intellect Disabilil* 2019; epub ahead of print.

30. Michie S, Van Stralen MM, West R. The behaviour change wheel: a new method for characterising and designing behaviour change interventions. *Implement Sci* 2011;**6**(1):42.

31. Zaal RJ, Ebbers S, Borms M, de Koning B, Mombarg E, Ooms P, et al. Medication review using a Systematic Tool to Reduce Inappropriate Prescribing (STRIP) in adults with an intellectual disability: A pilot study. *Res Dev Disabil* 2016;**55**:132-42.

32. Scheifes A, Egberts TC, Stolker JJ, Nijman HL, Heerdink ER. Structured medication review to improve pharmacotherapy in people with intellectual disability and behavioural problems. *J Appl Res Intellect Disabilil* 2016;**29**(4):346-55.

33. Bach LL, Lazzaretto DL, Young CF, Lofholm PW. Improving nursing home compliance via revised antipsychotic use survey tool. *Consult Pharm* 2017;**32**(4):228-38.

For peer review only - http://bmjopen.bmj.com/site/about/guidelines.xhtml

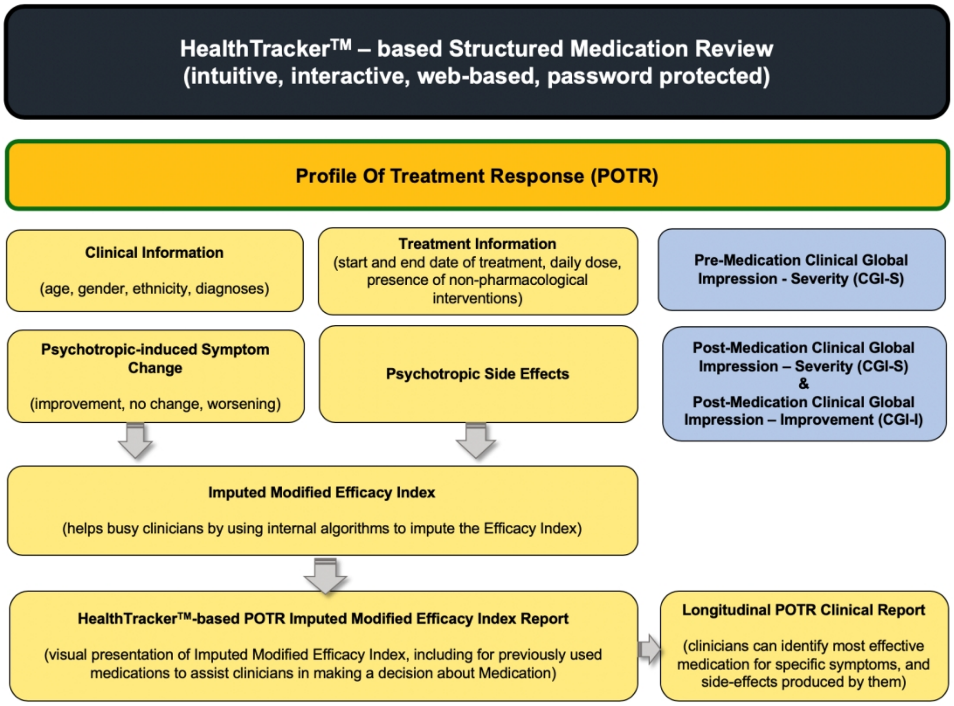

HealthTrackerTM-based structured medication review (HT-SMR)

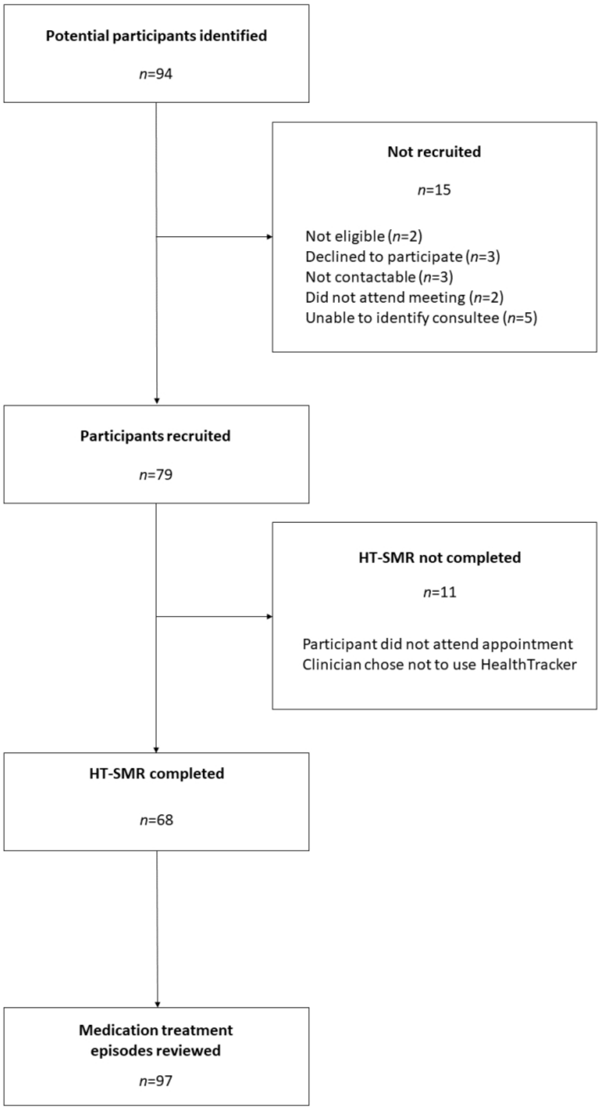

Participant flow

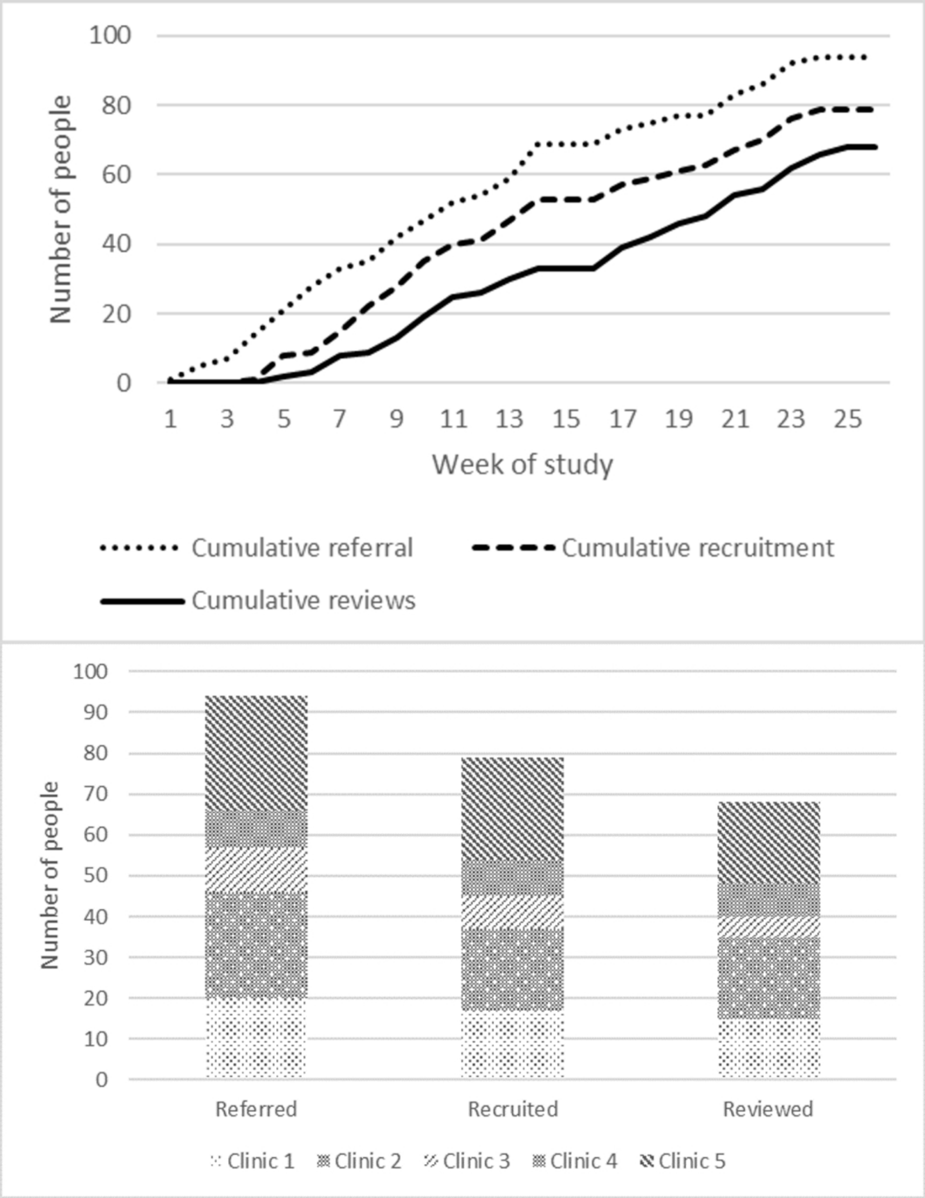

a) Rate of referral, recruitment, and use of the HT-SMR over the study period, and b) by participating clinical team

CONSORT 2010 checklist of information to include when reporting a pilot or feasibility trial*

Section/Topic	Item No	Checklist item	Reported on page No
**Title and abstract**			
	1a	Identification as a pilot or feasibility randomised trial in the title	1 & 3
	1b	Structured summary of pilot trial design, methods, results, and conclusions (for specific guidance see CONSORT abstract extension for pilot trials)	3 & 4
**Introduction**			
Background and objectives	2a	Scientific background and explanation of rationale for future definitive trial, and reasons for randomised pilot trial	7
	2b	Specific objectives or research questions for pilot trial	7
**Methods**			
Trial design	3a	Description of pilot trial design (such as parallel, factorial) including allocation ratio	7-10
	3b	Important changes to methods after pilot trial commencement (such as eligibility criteria), with reasons	N/A
Participants	4a	Eligibility criteria for participants	7
	4b	Settings and locations where the data were collected	7
	4c	How participants were identified and consented	7-8
Interventions	5	The interventions for each group with sufficient details to allow replication, including how and when they were actually administered	8-9
Outcomes	6a	Completely defined prespecified assessments or measurements to address each pilot trial objective specified in 2b, including how and when they were assessed	10-11
	6b	Any changes to pilot trial assessments or measurements after the pilot trial commenced, with reasons	N/A
	6c	If applicable, prespecified criteria used to judge whether, or how, to proceed with future definitive trial	N/A
Sample size	7a	Rationale for numbers in the pilot trial	10
	7b	When applicable, explanation of any interim analyses and stopping guidelines	N/A
Randomisation:			
Sequence generation	8a	Method used to generate the random allocation sequence	N/A
	8b	Type of randomisation(s); details of any restriction (such as blocking and block size)	N/A
Allocation concealment mechanism	9	Mechanism used to implement the random allocation sequence (such as sequentially numbered containers), describing any steps taken to conceal the sequence until interventions were assigned	N/A

For peer review only

Implementation	10	Who generated the random allocation sequence, who enrolled participants, and who assigned participants to interventions	N/A
Blinding	11a	If done, who was blinded after assignment to interventions (for example, participants, care providers, those assessing outcomes) and how	N/A
	11b	If relevant, description of the similarity of interventions	N/A
Statistical methods	12	Methods used to address each pilot trial objective whether qualitative or quantitative	10-11
**Results**			
Participant flow (a diagram is strongly recommended)	13a	For each group, the numbers of participants who were approached and/or assessed for eligibility, randomly assigned, received intended treatment, and were assessed for each objective	12 / figure 2
	13b	For each group, losses and exclusions after randomisation, together with reasons	12 / figure 2
Recruitment	14a	Dates defining the periods of recruitment and follow-up	12
	14b	Why the pilot trial ended or was stopped	N/A
Baseline data	15	A table showing baseline demographic and clinical characteristics for each group	13-14
Numbers analysed	16	For each objective, number of participants (denominator) included in each analysis. If relevant, these numbers should be by randomised group	12 / figure 2
Outcomes and estimation	17	For each objective, results including expressions of uncertainty (such as 95% confidence interval) for any estimates. If relevant, these results should be by randomised group	12
Ancillary analyses	18	Results of any other analyses performed that could be used to inform the future definitive trial	N/A
Harms	19	All important harms or unintended effects in each group (for specific guidance see CONSORT for harms)	13
	19a	If relevant, other important unintended consequences	N/A
**Discussion**			
Limitations	20	Pilot trial limitations, addressing sources of potential bias and remaining uncertainty about feasibility	23-25
Generalisability	21	Generalisability (applicability) of pilot trial methods and findings to future definitive trial and other studies	19-21
Interpretation	22	Interpretation consistent with pilot trial objectives and findings, balancing potential benefits and harms, and considering other relevant evidence	19-22
	22a	Implications for progression from pilot to future definitive trial, including any proposed amendments	22-23
**Other information**			
Registration	23	Registration number for pilot trial and name of trial registry	N/A
Protocol	24	Where the pilot trial protocol can be accessed, if available	N/A
Funding	25	Sources of funding and other support (such as supply of drugs), role of funders	26
	26	Ethical approval or approval by research review committee, confirmed with reference number	11-12

For peer review only

Citation: Eldridge SM, Chan CL, Campbell MJ, Bond CM, Hopewell S, Thabane L, et al. CONSORT 2010 statement: extension to randomised pilot and feasibility trials. BMJ. 2016;355.

*We strongly recommend reading this statement in conjunction with the CONSORT 2010, extension to randomised pilot and feasibility trials, Explanation and Elaboration for important clarifications on all the items. If relevant, we also recommend reading CONSORT extensions for cluster randomised trials, non-inferiority and equivalence trials, non-pharmacological treatments, herbal interventions, and pragmatic trials. Additional extensions are forthcoming: for those and for up to date references relevant to this checklist, see www.consort-statement.org.

For peer review only
